# Health-Related Quality of Life Outcomes With Two Different Starting Doses of Lenvatinib in Combination With Everolimus for Previously Treated Renal Cell Carcinoma

**DOI:** 10.1093/oncolo/oyac142

**Published:** 2023-01-18

**Authors:** Cristiane Bergerot, Sun Young Rha, Sumanta Pal, Piotr Koralewski, Daniil Stroyakovskiy, Boris Alekseev, Francis Parnis, Daniel Castellano, Jae Lyun Lee, Kaisa Sunela, Tudor Ciuleanu, Daniel Heng, Hilary Glen, Jinyi Wang, Lee Bennett, Janice Pan, Karen O’Hara, Javier Puente

**Affiliations:** Instituto Unity de Ensino e Pesquisa, Centro de Câncer de Brasília, Brasília, DF, Brazil; Yonsei Cancer Center, Yonsei University Health System, Seoul, Republic of Korea; Department of Medical Oncology & Therapeutics, City of Hope Comprehensive Cancer Center, Duarte, CA, USA; Szpital Specjalistyczny Ludwika Rydygiera, Kraków, Poland; Moscow City Oncology Hospital #62 of Moscow Healthcare Department, Moscow, Russia; P.A. Hertzen Moscow Oncological Research Institute, Moscow, Russia; University of Adelaide, Adelaide, South Australia, Australia; 12 de Octubre University Hospital, Madrid, Spain; Asan Medical Center, Seoul, Republic of Korea; University of Ulsan College of Medicine, Seoul, Republic of Korea; Department of Oncology, Tampere University Hospital, Tampere, Finland; Prof Dr Ion Chiricuta Cancer Institute and Iuliu Hatieganu University of Medicine and Pharmacy, Cluj-Napoca, Romania; Department of Oncology, Tom Baker Cancer Center, University of Calgary, Calgary, Alberta, Canada; Department of Medical Oncology, Beatson West of Scotland Cancer Centre, Glasgow, UK; RTI Health Solutions, Research Triangle Park, NC, USA; RTI Health Solutions, Research Triangle Park, NC, USA; Eisai, Inc., Nutley, NJ, USA; Eisai Europe Ltd., Hertfordshire, UK; Medical Oncology Department, Hospital Clínico San Carlos, Instituto de Investigación Sanitaria del Hospital Clínico San Carlos, Madrid, Spain

**Keywords:** EORTC QLQ-C30, FKSI-DRS, patient-reported outcomes, phase II, VEGF

## Abstract

**Background:**

Preserving health-related quality of life (HRQOL) is an important goal during renal cell carcinoma treatment. We report HRQOL outcomes from a phase II trial (NCT03173560).

**Patients and Methods:**

HRQOL data were collected during a multicenter, randomized, open-label phase II study comparing the safety and efficacy of 2 different starting doses of lenvatinib (18 mg vs. 14 mg daily) in combination with everolimus (5 mg daily), following one prior vascular endothelial growth factor–targeted treatment. HRQOL was measured using 3 different instruments—FKSI-DRS, EORTC QLQ-C30, and EQ-5D-3L—which were all secondary endpoints. Change from baseline was assessed using linear mixed-effects models. Deterioration events for time to deterioration (TTD) analyses were defined using established thresholds for minimally important differences in the change from baseline for each scale. TTD for each treatment arm was estimated using the Kaplan–Meier method.

**Results:**

Baseline characteristics of the 343 participants randomly assigned to 18 mg lenvatinib (*n* = 171) and 14 mg lenvatinib (*n* = 172) were well balanced. Least-squares mean estimates for change from baseline were favorable for the 18 mg group over the 14 mg group for the FKSI-DRS and most EORTC QLQ-C30 scales, but differences between treatments did not exceed the minimally important thresholds. Median TTD was longer among participants in the 18 mg group than those in the 14 mg group for most scales.

**Conclusions:**

Participants who received an 18 mg lenvatinib starting dose had favorable HRQOL scores and longer TTD on most scales compared with those who received a 14 mg starting dose.

## Teaser

Preserving health-related quality of life is an important goal during renal cell carcinoma treatment. This article reports health-related quality of life outcomes from a phase II trial (NCT03173560).

**Implications for Practice**

In phase II, open-label trial comparing 2 starting doses of lenvatinib (18 mg vs. 14 mg QD) in combination with everolimus (5 mg QD), the median time to definitive deterioration in most subscales of the FKSI-DRS, the EORTC QLQ-C30, and the EQ-5D-3L was longer among participants in the 18 mg group than those in the 14 mg group.

## Introduction

Renal cell carcinoma (RCC) is the most common type of kidney cancer, constituting 80-85% of primary renal neoplasms. RCC and the therapies used to treat patients with RCC are associated with a range of symptoms and treatment–related adverse events, which contribute to the burden of disease.^1^ Preserving health-related quality of life (HRQOL) is an important goal during RCC management, and thus RCC clinical trials should include assessments of patient-reported outcomes (PROs) to evaluate patients’ experiences with treatment.

Lenvatinib 18 mg daily (QD) in combination with everolimus 5 mg QD is approved in the United States, European Union, and other regions for the treatment of advanced RCC after one prior anti-angiogenic treatment. Study E7080-G000-218 (NCT03173560) is a multicenter, randomized, open-label (formerly double-blind), phase II study to assess the safety and efficacy of lenvatinib at 2 different starting doses (18 mg vs. 14 mg QD) in combination with everolimus (5 mg QD) following one prior vascular endothelial growth factor (VEGF)–targeted treatment. Results from Study 218 demonstrated that the 14-mg starting dose failed to demonstrate non-inferiority to the 18-mg starting dose, and had a comparable safety profile, thereby supporting the currently approved dosing paradigm.^2^

The objective of this analysis was to evaluate HRQOL and disease symptoms as secondary endpoints from Study 218. These outcomes were evaluated using the Functional Assessment of Cancer Therapy–Kidney Symptom Index–Disease-Related Symptoms (FKSI-DRS), the European Organization for Research and Treatment of Cancer Quality of Life Questionnaire for Patients with Cancer–Core 30 (EORTC QLQ-C30), and the EQ-5D 3 levels (EQ-5D-3L), which are validated for use in RCC and oncology.

## Patients and Methods

### Study Design and Participants

PRO data were collected during a multicenter, randomized, open-label, phase II study comparing the safety and efficacy of 2 different starting doses of lenvatinib (18 mg vs. 14 mg QD) in combination with everolimus (5 mg QD) following one prior VEGF-targeted treatment (NCT03173560). Eligible participants were adults (aged >18 years) with histological or cytological confirmation of predominant clear cell RCC, documented evidence of advanced RCC, and disease progression on or after VEGF-targeted treatment. All participants provided written informed consent.

Participants were randomly assigned to 1 of 2 treatment arms in a 1:1 ratio. In arm 1, patients were treated with lenvatinib 18 mg (orally, QD) plus everolimus 5 mg (orally, QD) (LEN18 + EVE). In arm 2, patients were treated with lenvatinib 14 mg (orally, QD) plus everolimus 5 mg (orally, QD) (LEN14 + EVE). Randomization was based on 2 stratification factors: Memorial Sloan Kettering Cancer Center (MSKCC) prognostic groups (favorable, intermediate, and poor risk) and whether participants had programmed death-1 (PD-1)/programmed death-ligand 1 (PD-L1) treatment (yes or no). Participants who were randomized to the LEN14 + EVE treatment group were required to undergo a dose increase to lenvatinib 18 mg on day 1 of cycle 2 if they did not experience any intolerable grade 2 or higher adverse events. Treatment was administered until disease progression, participant request or withdrawal of consent, unacceptable toxicity, or the end of the study.

PRO questionnaires were administered at baseline, on day 1 of each postbaseline treatment cycle after cycle 1, and at the end-of-treatment visit (up to 30 days after treatment discontinuation). Every effort was made to administer the PRO questionnaires prior to lenvatinib administration and before other assessments and procedures were conducted.

### Participant Demographic and Clinical Characteristics

Participants’ baseline demographic and clinical characteristics were collected prior to randomization into study arms. Characteristics included age, sex, Karnofsky Performance Status, MSKCC prognostic group, whether participants had a prior nephrectomy (yes or no), whether participants received prior PD-1/PD-L1 treatment (yes or no), and participants’ number of prior anticancer therapy regimens.

### PRO Measures

#### FKSI-DRS

The FKSI-DRS consists of 9 items that clinical experts and patients have indicated are important concepts for the treatment of advanced kidney cancer and that clinical experts have indicated are primarily disease-related and not treatment-related.^3^ The FKSI-DRS is used to assess symptoms including pain, fatigue, shortness of breath, fever, weight loss, coughing, and blood in the urine. Patients report the frequency/severity of each symptom using a 5-point Likert-type scale (0 = “not at all,” 1 = “a little bit,” 2 = “somewhat,” 3 = “quite a bit,” 4 = “very much”). A total score is calculated by numerically reversing and summing all item scores so that the total ranges from 0 to 36, with higher scores representing better HRQOL.

#### EORTC QLQ-C30

The EORTC QLQ-C30 is composed of 9 multiple-item scales and 6 single-item scales.^4^ The multiple-item scales include 5 functional scales (physical, role, emotional, cognitive, and social), 3 symptom scales (fatigue, nausea and vomiting, and pain), and a global health status/quality of life (QOL) score. The single-item scales include dyspnea, insomnia, appetite loss, constipation, diarrhea, and financial difficulties. Scores for all scales range from 0 to 100. For the global health status/QOL scale and the functional scales, higher scores indicate better QOL and functioning; for the symptom scales, higher scores indicate worse symptoms.

#### EQ-5D-3L

The EQ-5D is a general, preference-based PRO instrument that was developed to assess health outcomes for a wide variety of interventions on a common scale.^5^ It consists of a descriptive system of 5 questionnaire items and a visual analog scale (VAS). The instrument asks participants to rate their perceived health state today for 5 dimensions, including mobility, self-care, usual activities, pain/discomfort, and anxiety/depression. This study used the 3-level version of the EQ-5D (EQ-5D-3L). For this version, patients rate the items on a 3-point Likert-type scale, with 1 indicating “no problems,” 2 indicating “some problems,” and 3 indicating “extreme problems.” The VAS component of the EQ-5D measures current health status from 0 to 100, where 0 represents the “worst imaginable health state” and 100 represents the “best imaginable health state.”

The EQ-5D index was calculated by applying preference-based weights (tariffs) to the scores of the 5 health state domains. Index values can range from −1 to 1, with 0 representing a health state equivalent to death and 1 representing perfect health. Values less than 0 represent health states that are worse than death.^6^ Health states were mapped using United States time–trade-off (TTO) method tariffs^7^for North American participants, United Kingdom TTO method tariffs^8^ for both Western and Eastern European participants, and South Korean TTO method tariffs for participants from the Asia/Pacific region.^9^

### Statistical Analysis

Completion rates (ie, the percentage of participants who completed the instrument among all participants enrolled in the full analysis set at baseline) and compliance rates (ie, the percentage of participants who completed the instrument among all participants expected to complete the instrument) were summarized for each PRO instrument and scale by assessment timepoint and treatment arm.

The effect of treatment assignment on the change in PRO scores from baseline was assessed using mixed models. Specifically, mixed models with random coefficients were fitted using the change from baseline for each PRO score as the response variable. Each model included treatment, time, a time by treatment interaction term, baseline PRO score, and the 2 randomization stratification variables (MSKCC prognostic group and prior PD-1/PD-L1 treatment) along with patient-specific random intercept and slope terms. The covariance matrix for these random effects was assumed to be unstructured. The least-squares (LS) mean change from baseline for each treatment arm was estimated at each timepoint, along with an overall LS mean. The difference in LS means for the LEN18 + EVE versus LEN14 + EVE was also estimated.

The distribution of time to deterioration (TTD) and median TTD for each treatment arm were estimated using the Kaplan–Meier method. Both time to first deterioration^10^ and time to definitive deterioration^11^ were analyzed using established thresholds for minimally important differences in the change from baseline for each scale. Specifically, a deterioration event for each PRO was defined as a detrimental change in score, relative to baseline, that exceeded the minimally important difference (MID) for decline in the score. For the FKSI-DRS, the MID threshold was a decrease of 3 points or more.^3^ For the EORTC QLQ-C30 functional domain/QOL scores, the MID threshold was a decrease of 10 points or more, and for the EORTC QLQ-C30 symptom scores, the MID threshold was an increase of 10 points or more.^12^ For the EQ-5D, the MID threshold was a decrease of 0.08 points or more, and for the EQ-VAS, the MID threshold was a decrease of 7 points or more.^13^ Death due to any cause within 30 days of treatment discontinuation was considered a deterioration event. Participants without a deterioration event at the analysis cutoff date were censored at the date of the last PRO assessment. Cox models stratified by the randomized stratification variables were fit for each score; hazard ratios (HRs) and 95% CIs were estimated to compare the 2 treatment arms.

All statistical tests and CIs have an associated alpha level of 0.05. No adjustments for multiple testing or estimation were used, so all *P* values and CIs should be considered nominal and descriptive in nature. All analyses were performed using SAS version 9.4 (SAS Institute, Cary, NC).

## Results

### Baseline Participant Characteristics

Participant demographic and clinical characteristics were balanced between the LEN18 + EVE (*n* = 171) and LEN14 + EVE (*n* = 172) study arms (Table 1).

**Table 1.** Baseline participant characteristics

Characteristic	Lenvatinib 18 mg + everolimus 5 mg (*n* = 171)	Lenvatinib 14 mg + everolimus 5 mg (*n* = 172)
Age, years		
Median (range)	62 (35-87)	61 (28-82)
Sex, *n* (%)		
Male	129 (75.4)	133 (77.3)
Karnofsky performance status, *n* (%)		
Score ≥90	124 (72.5)	128 (74.4)
MSKCC prognostic group, *n* (%)		
Favorable risk	50 (29.2)	49 (28.5)
Intermediate risk	90 (52.6)	93 (54.1)
Poor risk	31 (18.1)	30 (17.4)
Prior nephrectomy, *n* (%)		
Yes	140 (81.9)	144 (83.7)
Prior PD-1/PD-L1 treatment, *n* (%)		
Yes	41 (24)	49 (28.5)
Number of prior anticancer therapy regimens, *n* (%)		
1	140 (81.9)	129 (75)
2	29 (17)	38 (22.1)
≥3	2 (1.2)	5 (2.9)

Abbreviations: MSKCC, Memorial Sloan Kettering Cancer Center; PD-1, programmed death-1; PD-L1, programmed death-ligand 1.

### Completion and Compliance Rates

Completion rates for all PRO instruments were generally similar between treatment arms, with rates for completion of any instrument declining below 50% by cycle 10 in both groups. Less than half of enrolled participants in both treatment groups had a valid score at the off-treatment visit. Compliance was high (> 90%) in both groups during treatment and was lower at the off-treatment visit, where compliance for any instrument was 77.3% in the LEN18 + EVE group and 80.2% in the LEN14 + EVE group.

### Longitudinal Change From Baseline

**Figure 1.**
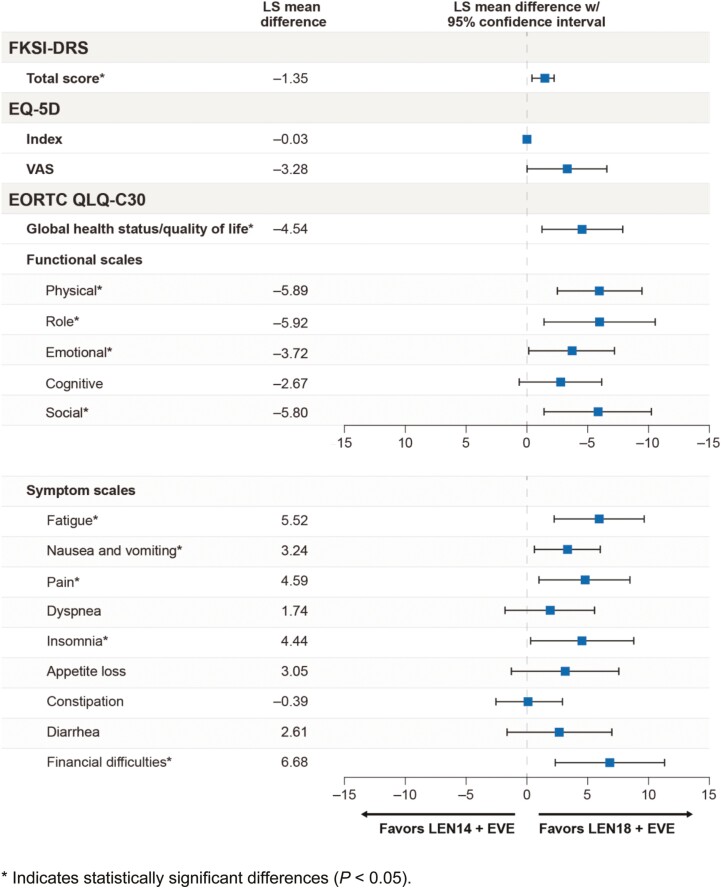
Overall least-squares mean differences between treatments. *Indicates statistically significant differences (*P* < .05).

Abbreviations: FKSI-DRS, Functional Assessment of Cancer Therapy–Kidney Symptom Index–Disease-Related Symptoms; LEN14 + EVE, lenvatinib 14 mg + everolimus 5 mg; LEN18 + EVE, lenvatinib 18 mg + everolimus 5 mg; QLQ-C30, Quality of Life Questionnaire for Patients with Cancer–Core 30; VAS, visual analog scale.

Overall LS mean differences estimated at the mean follow-up time (approximately 34 weeks [241 days], with 95% CIs) between the treatment arms for each scale are shown in Fig. 1. Differences in the overall LS means, which were nominally statistically significant, were seen for the FKSI-DRS total and the EORTC QLQ-C30 global health status/QOL, physical functioning, role functioning, emotional functioning, social functioning, fatigue, nausea and vomiting, pain, insomnia, and financial difficulties scales. There were no statistically notable differences seen for the cognitive functional scale, 3 of the gastrointestinal symptom scales (appetite loss, constipation, diarrhea), or dyspnea.

EORTC QLQ-C30 symptom scales are plotted in reverse, as higher values indicate worse symptoms.

### Time to Deterioration

#### Time to First Deterioration

**Figure 2.**
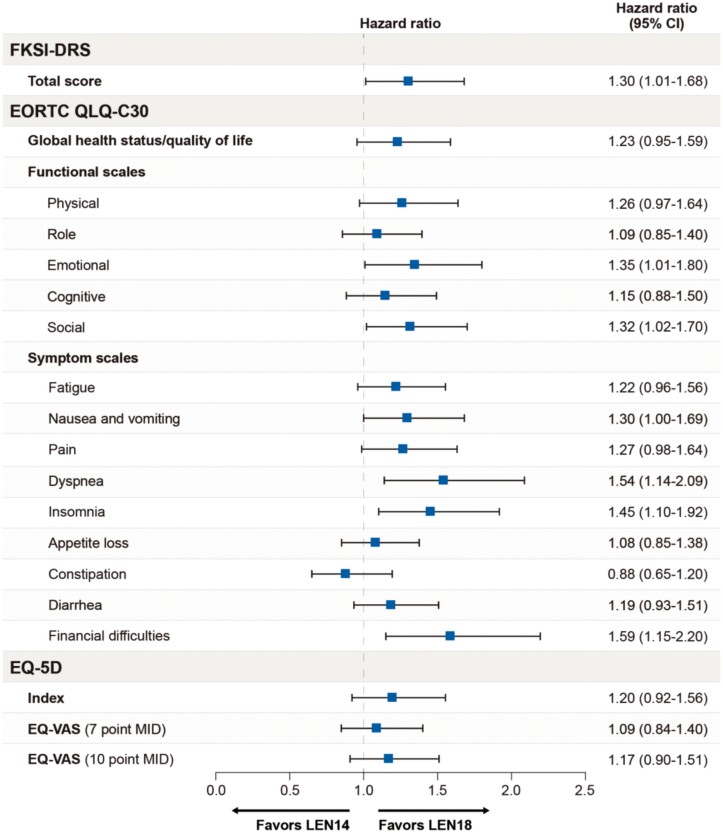
Time to first deterioration: hazard ratios.

Abbreviations: CI, confidence interval; EORTC QLQ-C30, European Organization for Research and Treatment of Cancer Quality of Life Questionnaire for Patients with Cancer–Core 30; FKSI-DRS, Functional Assessment of Cancer Therapy–Kidney Symptom Index–Disease-Related Symptoms; LEN14, lenvatinib 14 mg; LEN18, lenvatinib 18 mg; MID, minimally important difference; QOL, quality of life.

**Figure 3.**
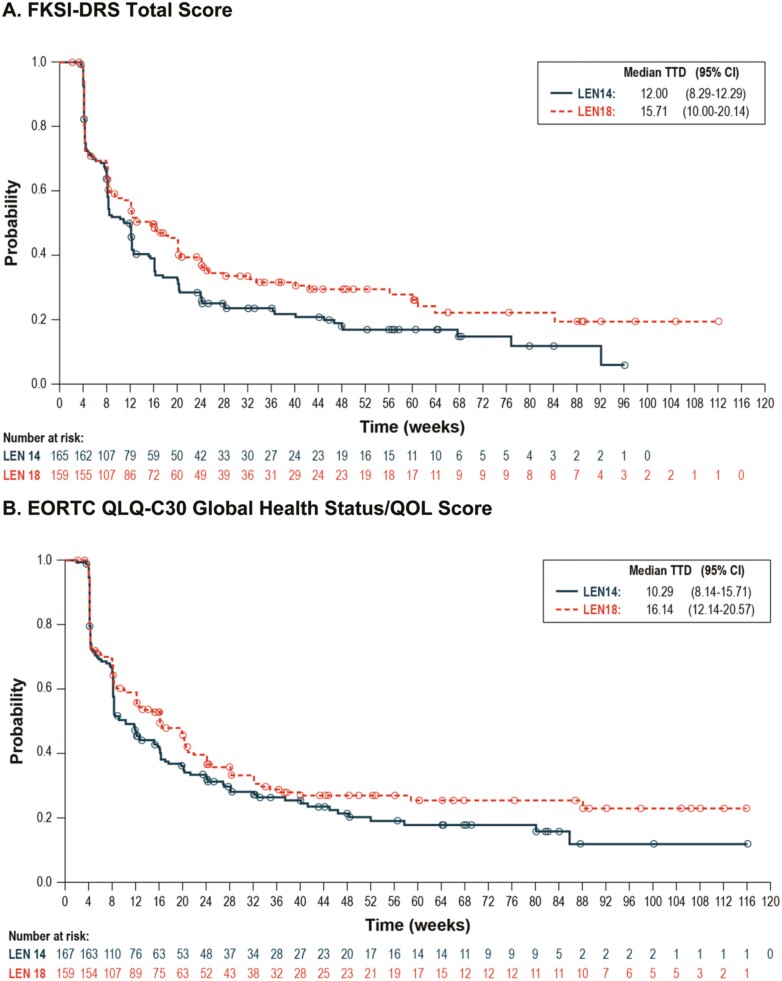

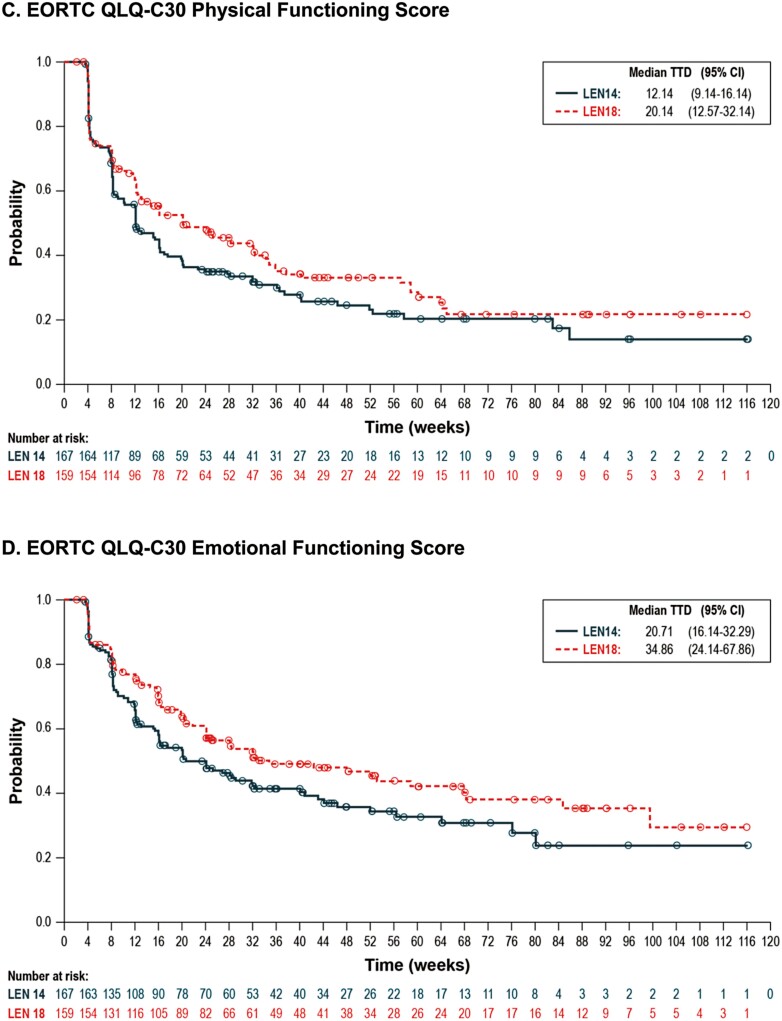

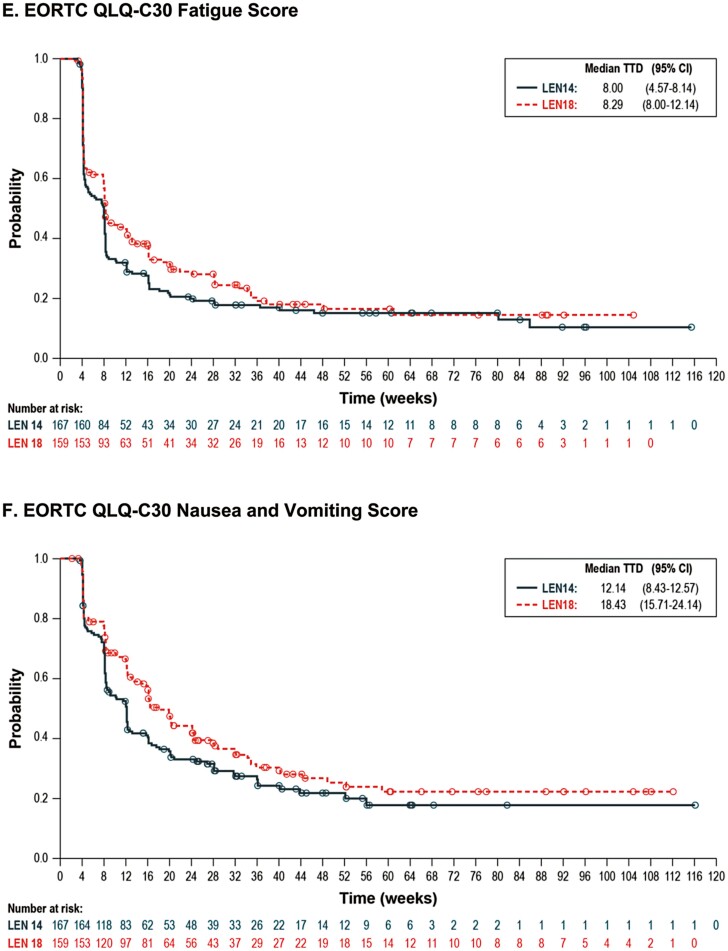
Kaplan–Meier plots of time to first deterioration. **A.** FKSI-DRS total score. **B.** EORTC QLQ-C30 Global Health Status/QOL Score. **C.** EORTC QLQ-C30 Physical Functioning Score. **D.** EORTC QLQ-C30 Emotional Functioning Score. **E.** EORTC QLQ-C30 Fatigue Score. **F.** EORTC QLQ-C30 Nausea and Vomiting Score.

Abbreviations: CI, confidence interval; EORTC QLQ-C30, European Organization for Research and Treatment of Cancer Quality of Life Questionnaire for Patients with Cancer–Core 30; FKSI-DRS, Functional Assessment of Cancer Therapy–Kidney Symptom Index–Disease-Related Symptoms; LEN14, lenvatinib 14 mg; LEN18, lenvatinib 18 mg; QOL, quality of life; TTD, time to deterioration.

The median time to first deterioration was shorter for the LEN14 + EVE treatment group than for the LEN18 + EVE treatment group for nearly all scales, with the exception of EORTC QLQ-C30 constipation and role functioning. The median time to first deterioration was nominally statistically significantly shorter for the LEN14 + EVE treatment group than for the LEN18 + EVE treatment group for the FKSI-DRS scale and the EORTC QLQ-C30 emotional functioning, social functioning, dyspnea, insomnia, and financial difficulties scales. HRs with 95% CIs comparing LEN14+EVE with LEN18+EVE are shown in Figs. 2 and 3 and Supplementary Fig. S1 (in the Supplementary Appendix) present Kaplan–Meier distributions of median time to first deterioration by treatment group. Scales with nominally significant 95% CIs for HRs included the FKSI-DRS total score and the EORTC QLQ-C30 emotional functioning, social functioning, dyspnea, insomnia, and financial difficulties scales. For all of these scales, the HRs favored the LEN18+EVE group.

#### Time to Definitive Deterioration

**Figure 4.**
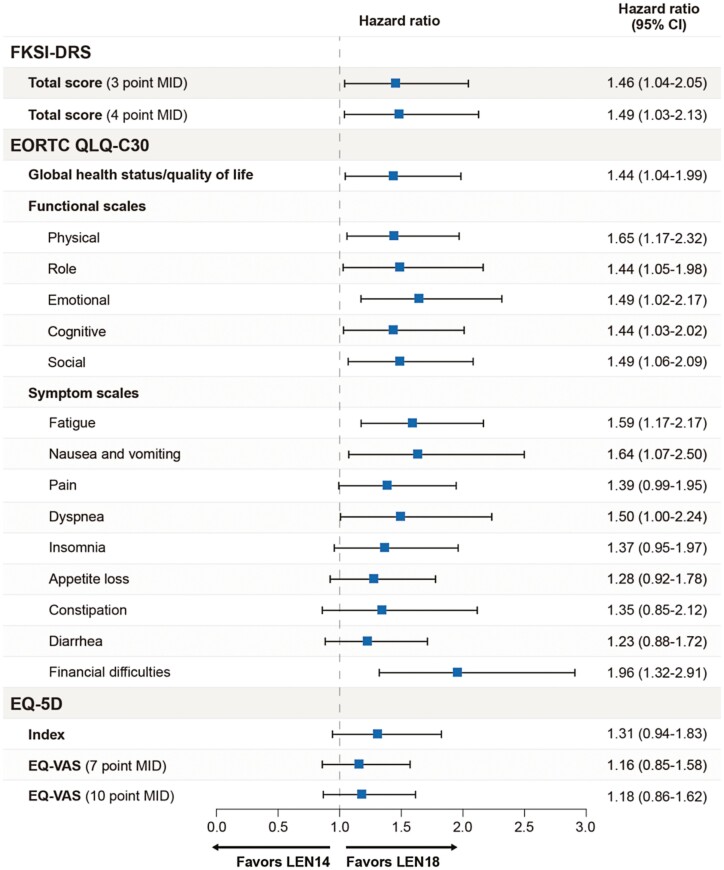
Time to definitive deterioration: hazard ratios.

Abbreviations: CI, confidence interval; EORTC QLQ-C30, European Organization for Research and Treatment of Cancer Quality of Life Questionnaire for Patients with Cancer–Core 30; FKSI-DRS, Functional Assessment of Cancer Therapy–Kidney Symptom Index–Disease-Related Symptoms; LEN14, lenvatinib 14 mg; LEN18, lenvatinib 18 mg; MID, minimally important difference; QOL, quality of life; VAS, visual analog scale.

**Figure 5.**
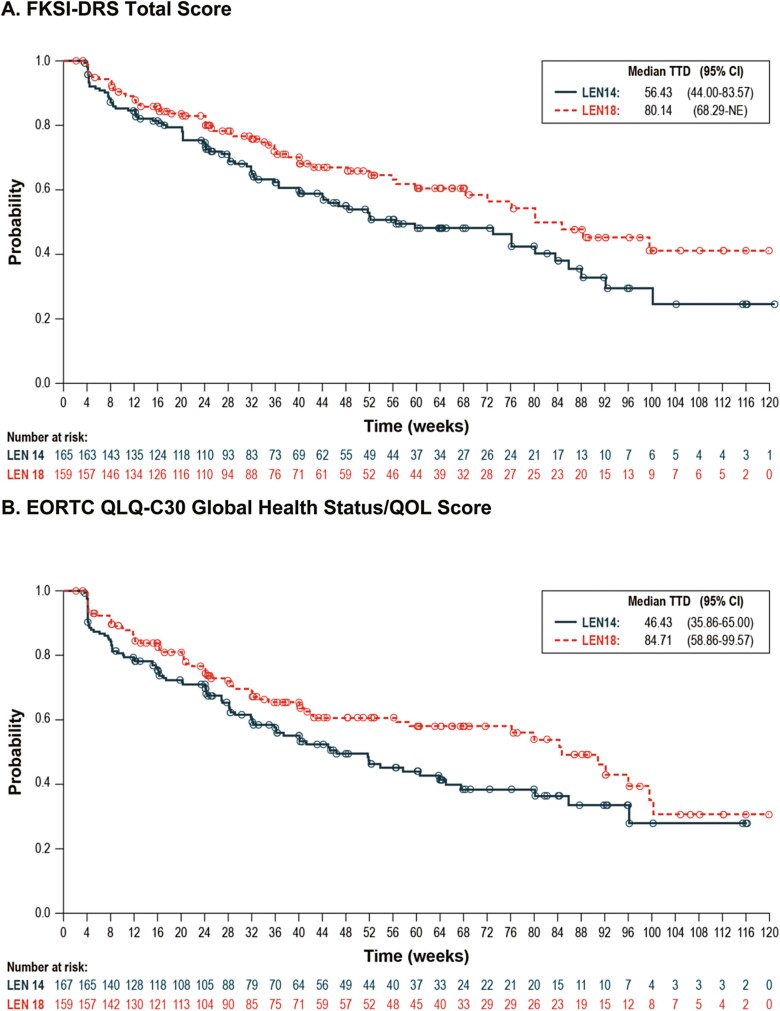

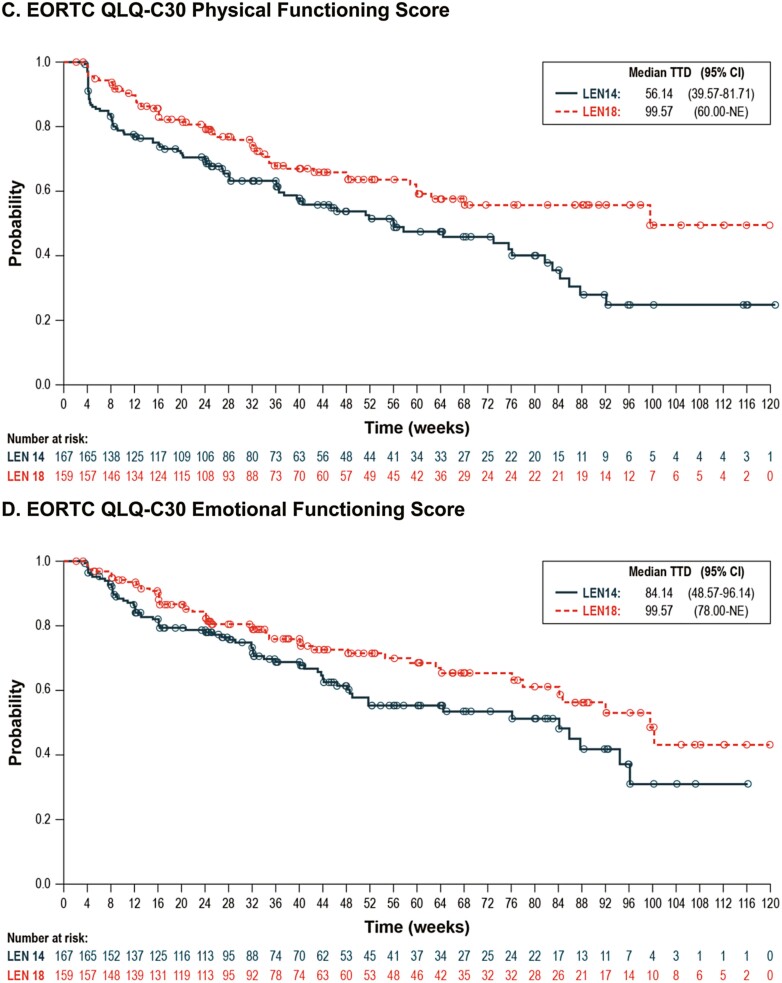

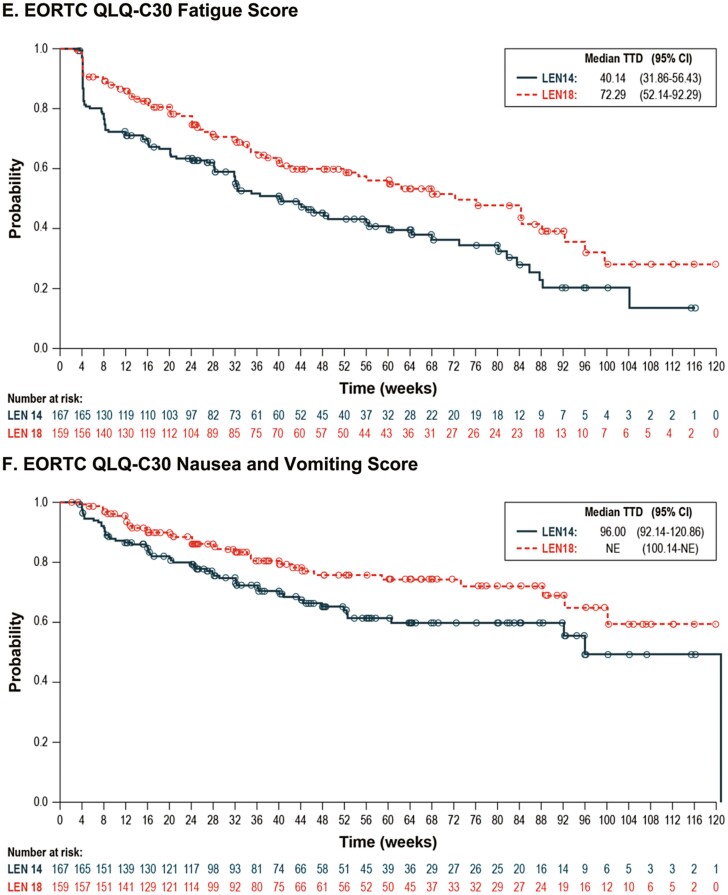
Kaplan–Meier plots of time to definitive deterioration. **A.** FKSI-DRS Total Score. **B.** EORTC QLQ-C30 Global Health Status/QOL Score. **C.** EORTC QLQ-C30 Physical Functioning Score. **D.** EORTC QLQ-C30 Emotional Functioning Score. **E.** EORTC QLQ-C30 Fatigue Score. **F.** EORTC QLQ-C30 Nausea and Vomiting Score.

Abbreviations: CI, confidence interval; EORTC QLQ-C30, European Organization for Research and Treatment of Cancer Quality of Life Questionnaire for Patients with Cancer–Core 30; FKSI-DRS, Functional Assessment of Cancer Therapy–Kidney Symptom Index–Disease-Related Symptoms; LEN14, lenvatinib 14 mg; LEN18, lenvatinib 18 mg; NE, not estimable; QOL, quality of life; TuDD, time until definitive dseterioration.

The median time to definitive deterioration was shorter for the LEN14 + EVE treatment group than for the LEN18 + EVE treatment group across all domains. HRs with 95% CIs comparing LEN14+EVE with LEN18+EVE are shown in Figs. 4 and 5 and Supplementary Fig. S2 (in the Supplementary Appendix) present Kaplan–Meier distributions of median time to definitive deterioration by treatment group. Scales with statistically significant 95% CIs for HRs comparing the 2 treatment groups included the FKSI-DRS total score and EORTC QLQ-C30 global health status/QOL, physical functioning, role functioning, emotional functioning, cognitive functioning, social functioning, fatigue, nausea and vomiting, and financial difficulties.

## Discussion

This analysis of PRO data from a prospective RCC clinical trial provided valuable insight into how RCC treatment starting dosage can impact patients’ HRQOL and disease symptoms differentially. The clinical objective of the trial was to evaluate whether the 14 mg starting dose of lenvatinib has comparable efficacy with improved safety when compared with the currently approved 18 mg starting dose of lenvatinib when either dose is combined with 5 mg everolimus QD. For this reason, it is also important to determine if the treatments provide comparable participant experiences with regard to HRQOL and disease burden. However, no statistical hypotheses were prespecified for these outcomes, so the results must be viewed as descriptive in nature.

Based on the longitudinal analyses of change from baseline scores, there were nominally significant overall differences in favor of the 18 mg lenvatinib treatment group for the FKSI-DRS and most EORTC QLQ-C30 domains. Although none of the differences rose to the level of being clinically meaningful, these results do suggest that participants in the 18 mg lenvatinib group had slightly better HRQOL and less severe treatment-related symptoms at the average follow-up time. Gastrointestinal symptoms and dyspnea were the exceptions, having approximately equal average severity in the 2 treatment arms.

Deterioration was the most frequently observed outcome for all HRQOL scales, and the distribution of outcomes was similar for most scales between the 2 treatment arms. The only exceptions were the EORTC QLQ-C30 insomnia and financial difficulties scales; for both, a larger proportion of participants in the lenvatinib 14 mg arm experienced deterioration compared with the lenvatinib 18 mg arm. Potential drivers of differences in these outcomes between dosage arms are unknown; in particular, financial difficulties as measured by the EORTC QLQ-C30 have been found to be associated with worse HRQOL and survival outcomes in patients with multiple tumor types.^14^

The TTD analyses are consistent with the longitudinal analysis with regard to longer maintenance of HRQOL and symptom control within the lenvatinib 18 mg treatment group, particularly for definitive deterioration. The time to symptom deterioration was not significantly different for diarrhea, appetite loss, and constipation; these results, when viewed with the changes in scores for these domains over time, suggest that the 2 dosages of lenvatinib have similar effects on these gastrointestinal symptoms.

Some limitations of these analyses should be considered. First, due to the open-label study design, the findings should be interpreted with caution. All patients were analyzed as part of the group to which they were randomized. Those who escalated from 14 to 18 mg were part of the 14 mg arm for analysis; we did not quantify the number of patients who escalated or adjust the analyses to account for the dose escalation. In addition, the analyses did not account for dose reduction, dose modification, or treatment duration in either arm, each of which may in turn affect HRQOL or TTD. Missing data were assumed to be missing at random, and the analyses included no adjustment for multiplicity.

## Conclusions

These results indicate that HRQOL and disease symptoms for participants in the 14 mg QD lenvatinib treatment group were similar to or slightly worse than those for participants in the 18 mg QD lenvatinib treatment group. These findings support the approved treatment of the 18 mg QD starting dose of lenvatinib in combination with everolimus as an effective treatment option for patients with RCC following one prior VEGF-targeted treatment while maintaining QOL. Efficacy and safety data show that disease is better controlled with the approved 18 mg starting dose without significant clinical deterioration and that patients may derive some QOL benefit at this dose level.

## Supplementary Material

oyac142_suppl_Supplementary_Material

## Data Availability

The source data for this article are considered both commercially confidential and Health Insurance Portability and Accountability Act confidential; therefore, the data holders (Eisai) do not intend to publicly post or share the source data. Eisai may, however, consider requests on a case-by-case basis if contacted by an individual researcher (access will be permitted after signature of a data-access agreement).

## References

[1] Cella D. Quality of life in patients with metastatic renal cell carcinoma: the importance of patient-reported outcomes. Cancer Treat Rev. 2009;35(8):733-737. 10.1016/j.ctrv.2009.07.003. 19699588

[2] Pal S , Puente J, Heng DYC, et al. Phase 2 trial of lenvatinib at 2 starting doses + everolimus in renal cell carcinoma. Presented at: International Kidney Cancer Symposium 2020; November 6–7, 2020.

[3] Cella D , Yount S, Brucker PS, et al. Development and validation of a scale to measure disease-related symptoms of kidney cancer. Value Health. 2007;10(4):285-293. 10.1111/j.1524-4733.2007.00183.x. 17645683

[4] Aaronson NK , Ahmedzai S, Bergman B, et al. The European Organization for Research and Treatment of Cancer QLQ-C30: a quality-of-life instrument for use in international clinical trials in oncology. J Natl Cancer Inst. 1993;85(5):365-376. 10.1093/jnci/85.5.365 . 8433390

[5] EuroQol Research Foundation. EQ-5D-3L User Guide. Version 6. 2018. https://euroqol.org/wp-content/uploads/2018/12/EQ-5D-3L-User-Guide_version-6.0.pdf. Accessed July 1, 2020.

[6] Bernfort L , Gerdle B, Husberg M, Levin LA. People in states worse than dead according to the EQ-5D UK value set: would they rather be dead? Qual Life Res. 2018 ;27(7):1827-1833. 10.1007/s11136-018-1848-x. 29616427 PMC5997722

[7] Shaw JW , Johnson JA, Coons SJ. US valuation of the EQ-5D health states: development and testing of the D1 valuation model. Med Care. 2005;43(3):203-220. 15725977 10.1097/00005650-200503000-00003

[8] Dolan P. Modeling valuations for EuroQol health states. Med Care. 1997 ;35(11):1095-1108. 10.1097/00005650-199711000-00002. 9366889

[9] Lee YK , Nam HS, Chuang LH, et al. South Korean time trade-off values for EQ-5D health states: modeling with observed values for 101 health states. Value Health. 2009 ;12(8):1187-1193. 19659703 10.1111/j.1524-4733.2009.00579.x

[10] Hamidou Z , Dabakuyo TS, Mercier M, et al. Time to deterioration in quality of life score as a modality of longitudinal analysis in patients with breast cancer. Oncologist. 2011;16(10):1458-1468. 10.1634/theoncologist.2011-0085. 21948650 PMC3228064

[11] Bonnetain F , Dahan L, Maillard E, et al. Time until definitive quality of life score deterioration as a means of longitudinal analysis for treatment trials in patients with metastatic pancreatic adenocarcinoma. Eur J Cancer. 2010 ;46(15):2753-2762. 10.1016/j.ejca.2010.07.023. 20724140

[12] Osoba D , Rodrigues G, Myles J, et al. Interpreting the significance of changes in health-related quality-of-life scores. J Clin Oncol. 1998;16(1):139-144. 10.1200/JCO.1998.16.1.139. 9440735

[13] Pickard AS , Neary MP, Cella D. Estimation of minimally important differences in EQ-5D utility and VAS scores in cancer. Health Qual Life Outcomes. 2007;5:70. 10.1186/1477-7525-5-70. 18154669 PMC2248572

[14] Perrone F , Jommi C, Di Maio M, et al. The association of financial difficulties with clinical outcomes in cancer patients: secondary analysis of 16 academic prospective clinical trials conducted in Italy. Ann Oncol. 2016;27(12):2224-2229. 10.1093/annonc/mdw433. 27789469

