# Supplementary material for: Health-Related Quality of Life Outcomes With Two Different Starting Doses of Lenvatinib in Combination With Everolimus for Previously Treated Renal Cell Carcinoma
Source: Oncologist. 2023 Jan 18;28(1):59–71. doi: 10.1093/oncolo/oyac142 (PMC9847563; doi:10.1093/oncolo/oyac142)

# Supplemental Appendix

Figure S1. Kaplan-Meier Plots of Time to First Deterioration


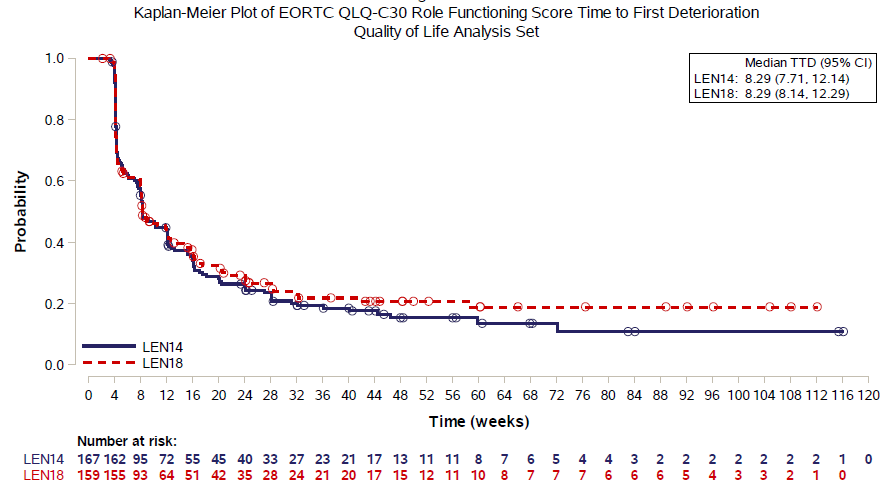


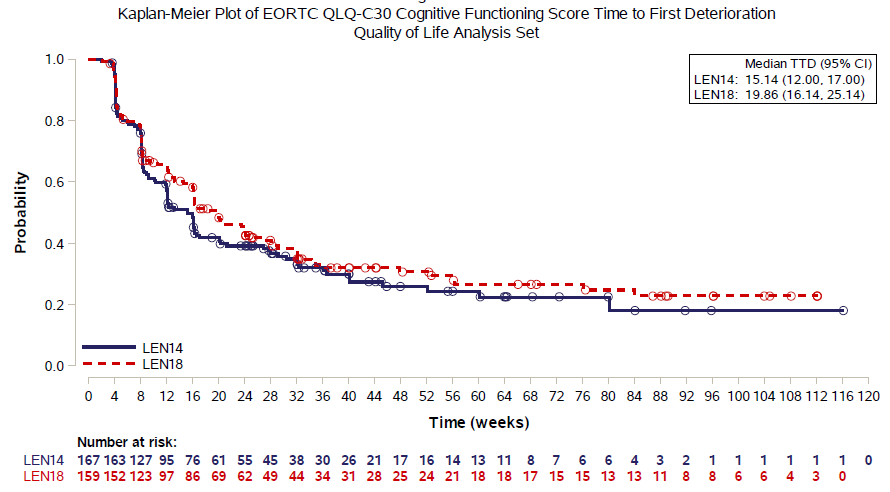


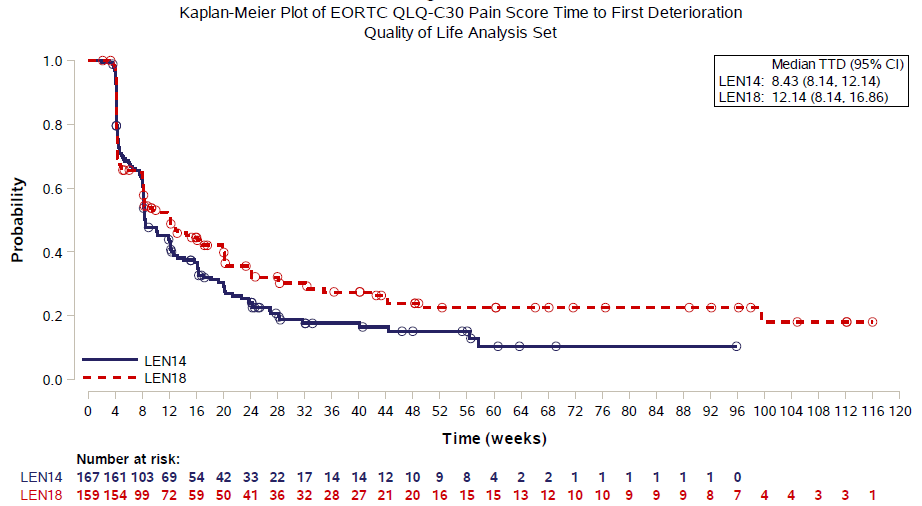


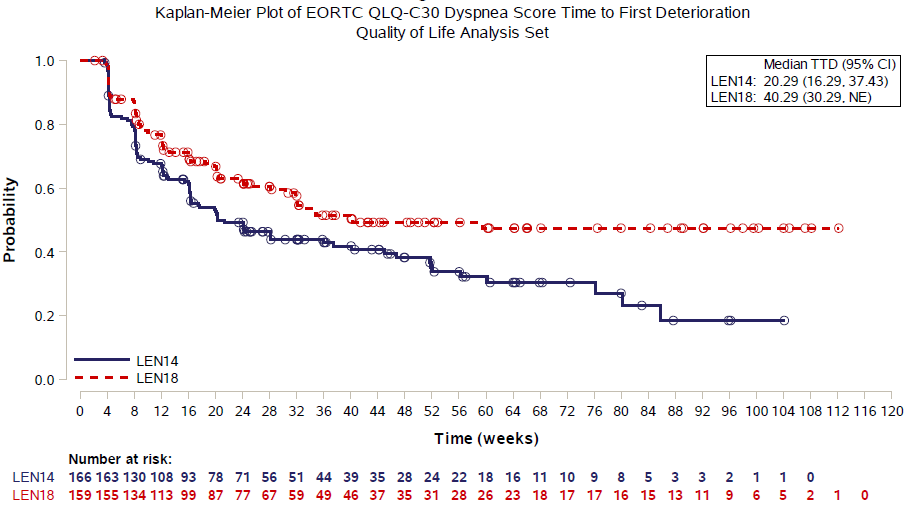


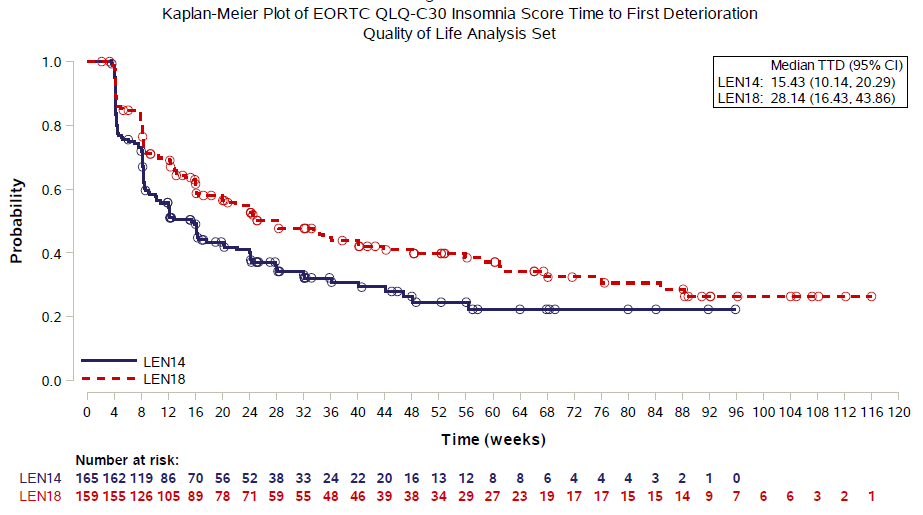


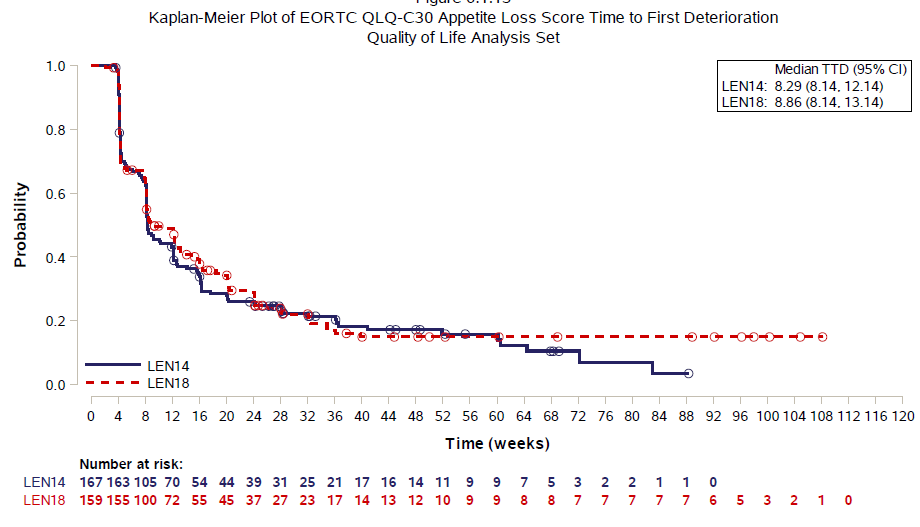


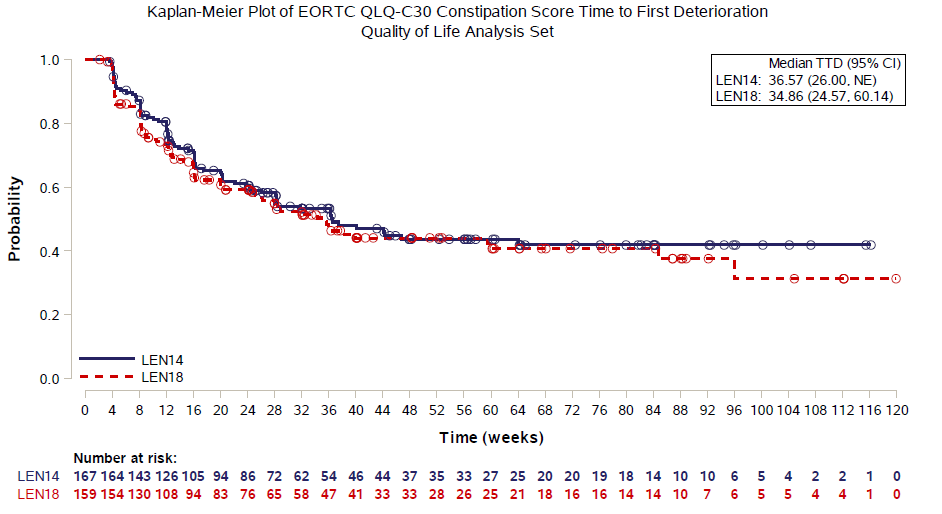


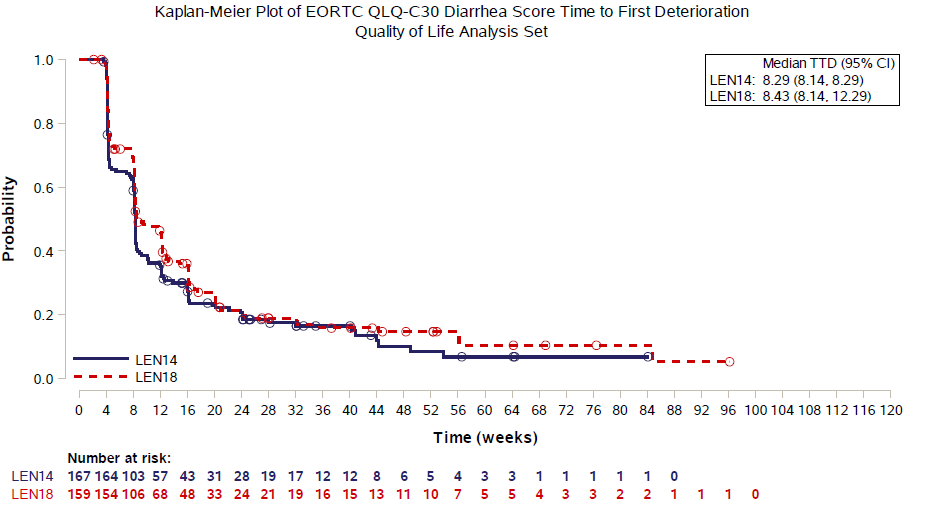


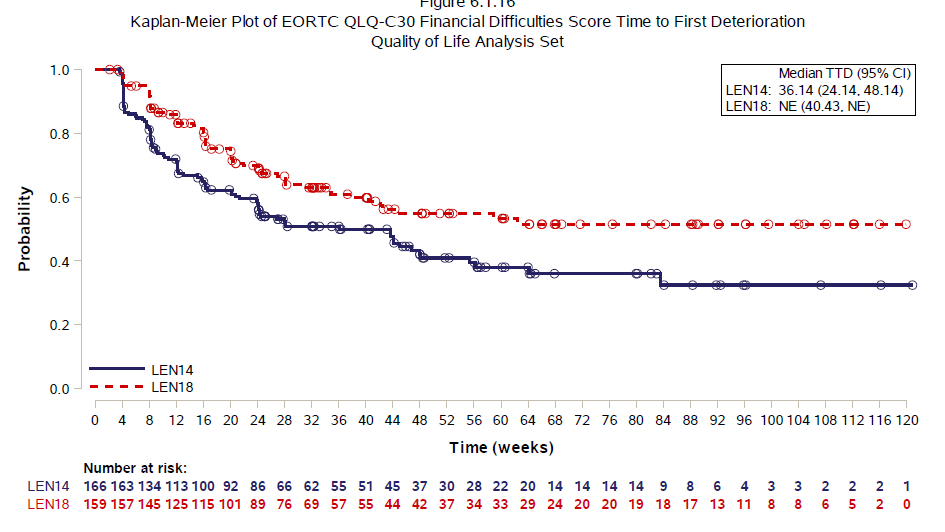


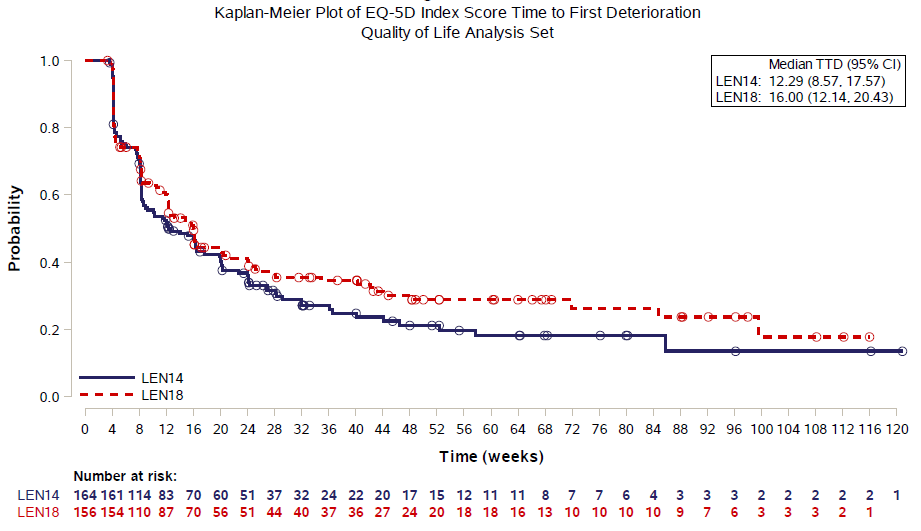


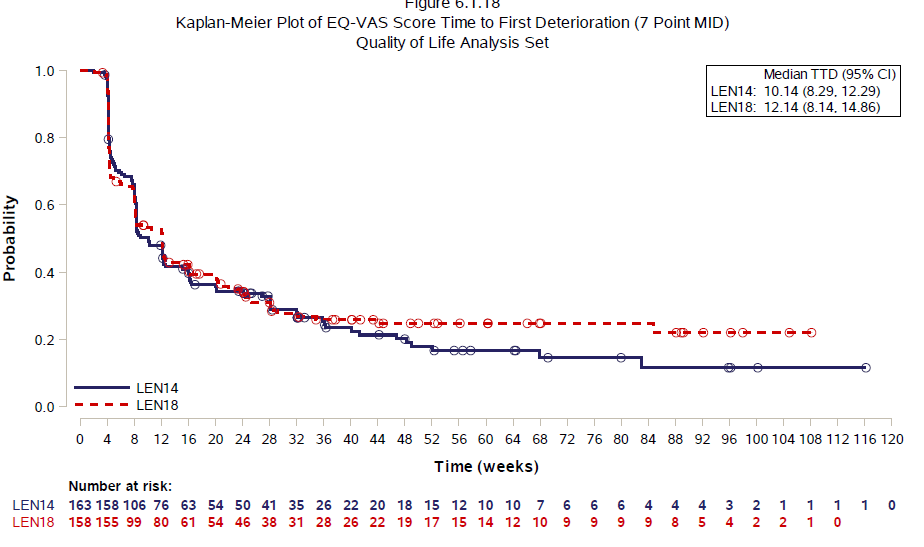


Figure S2. Kaplan-Meier Plots of Time to Definitive Deterioration


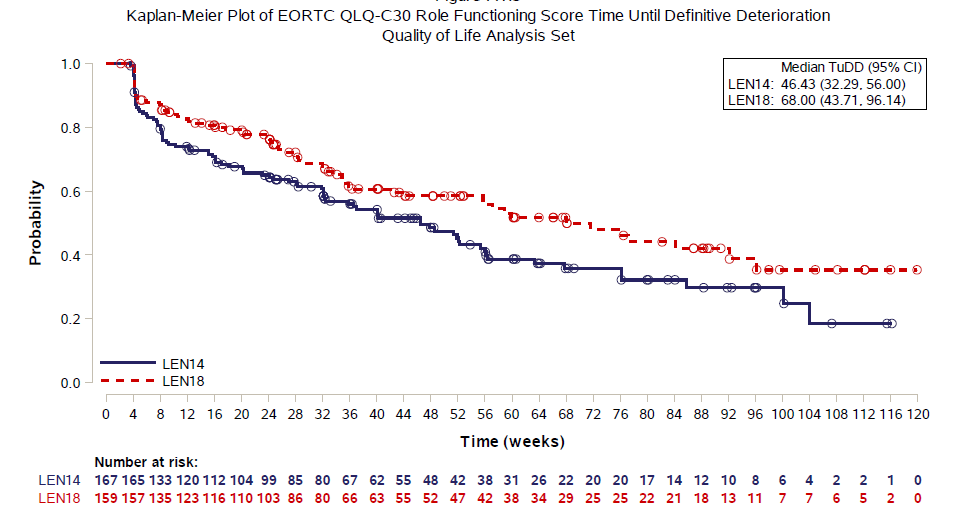


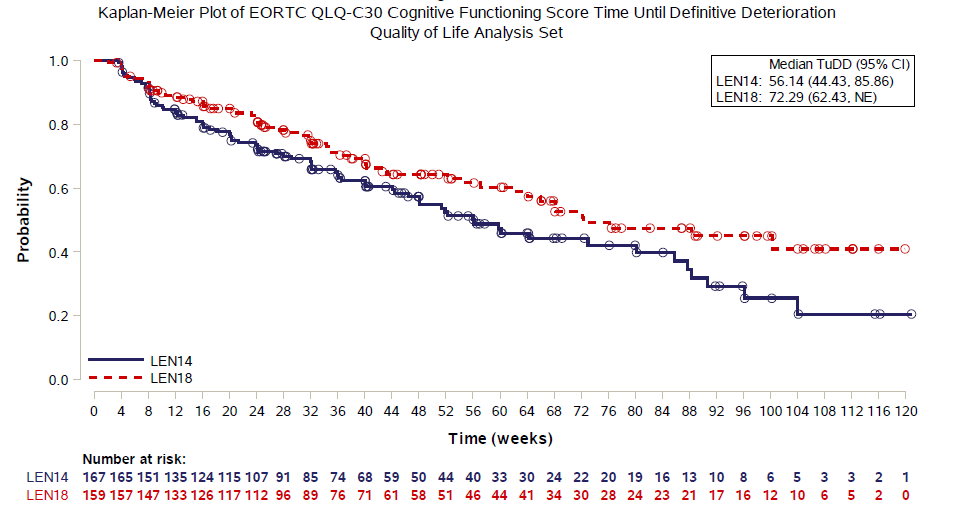


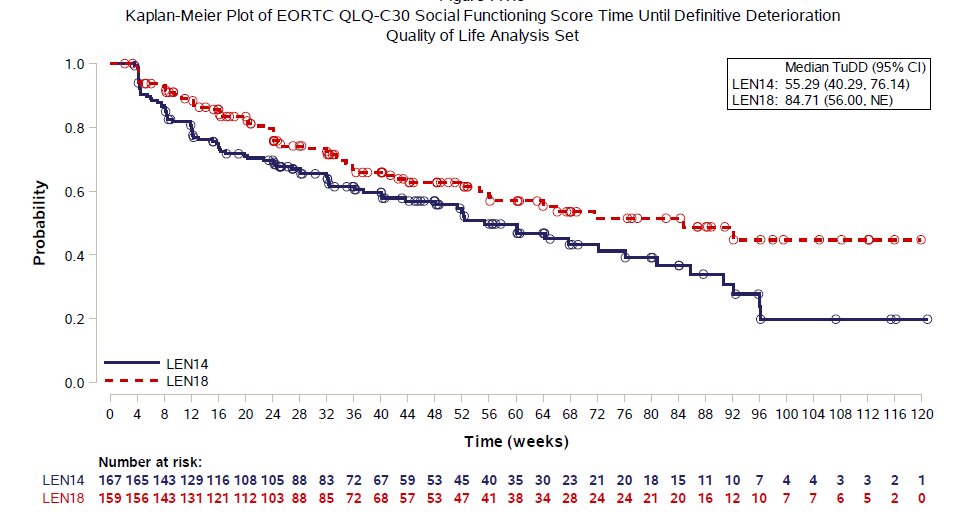


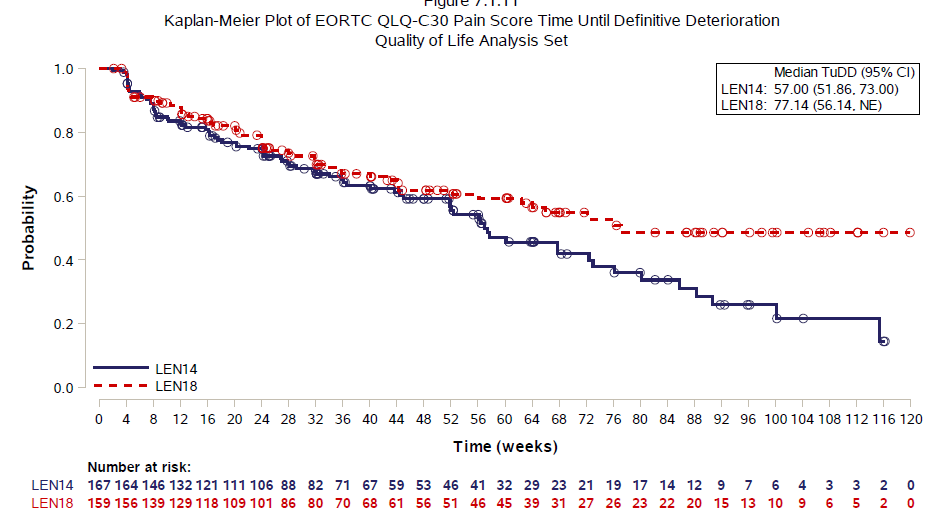


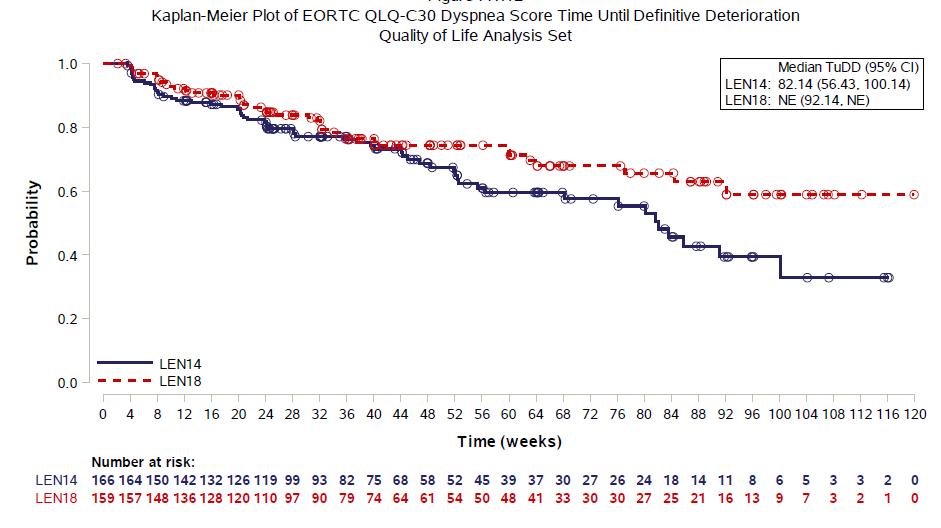


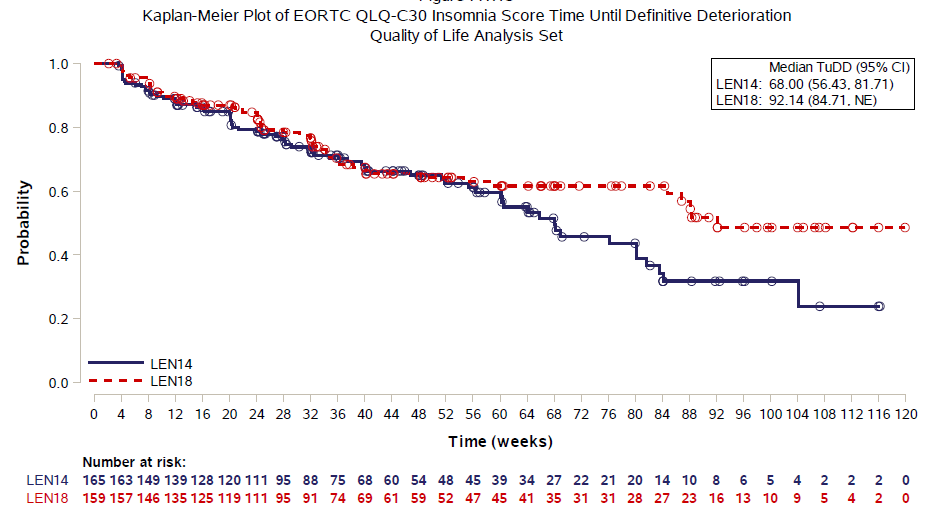


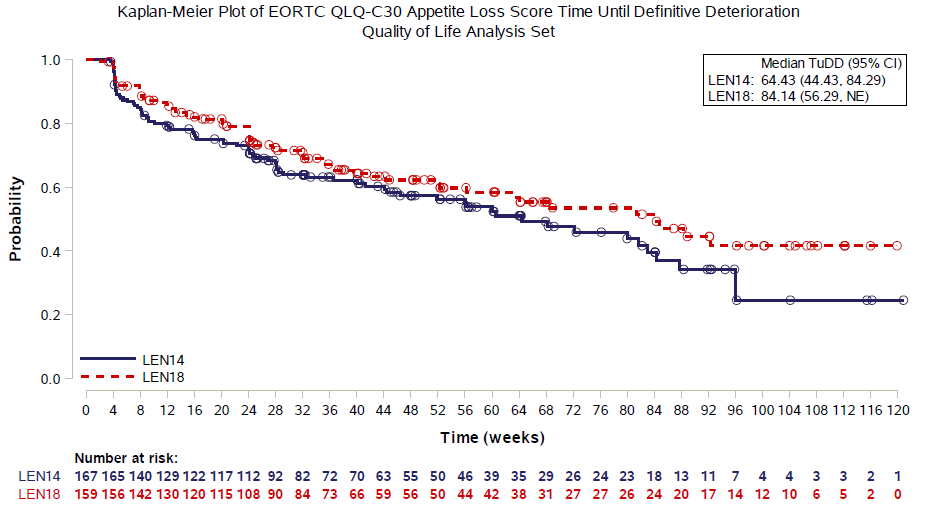


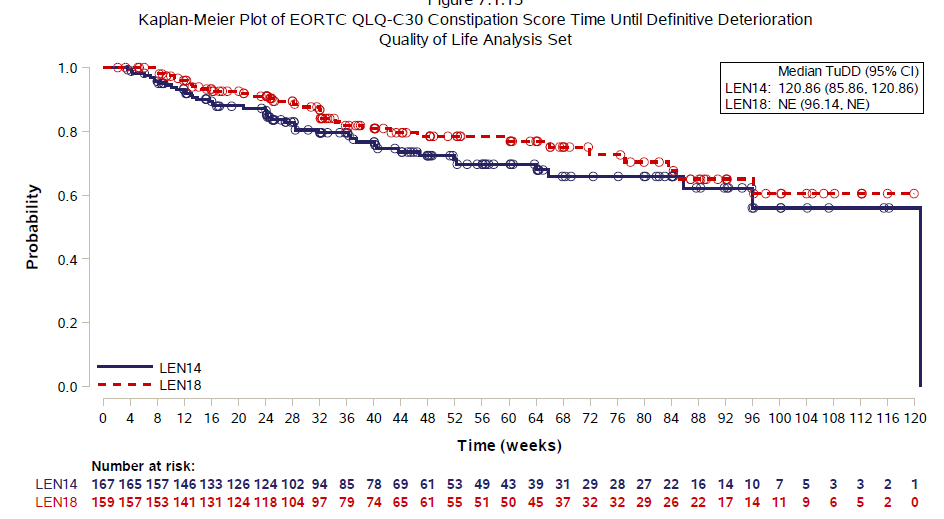


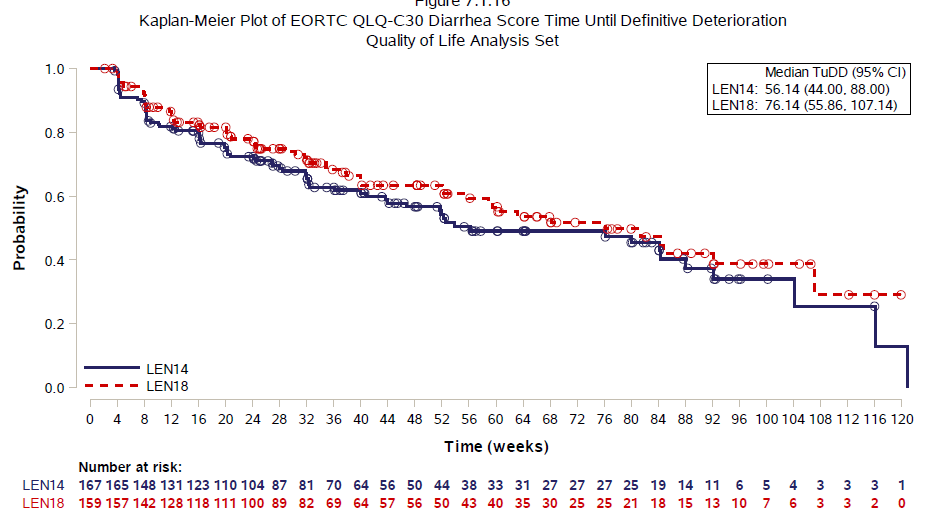


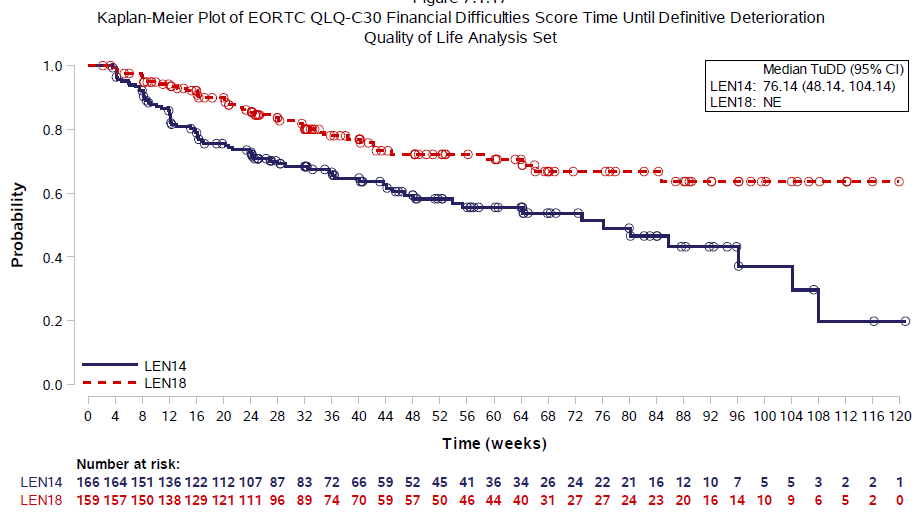


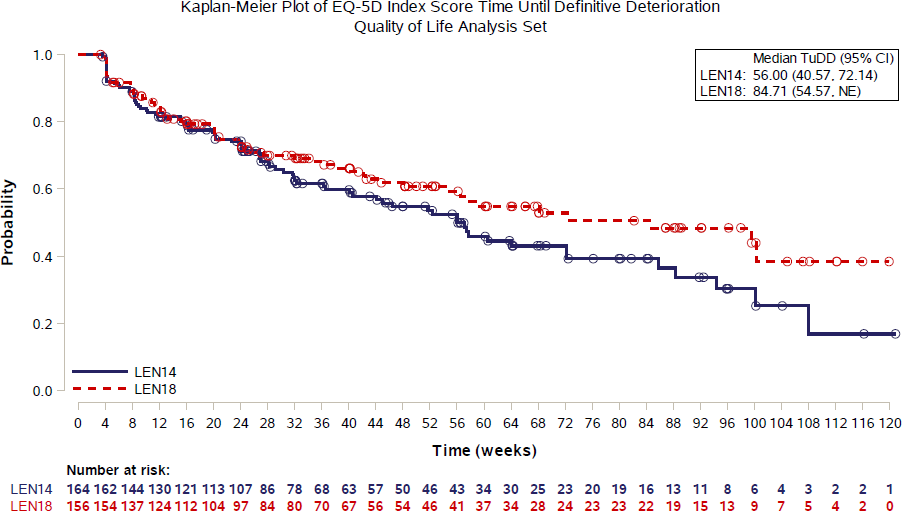


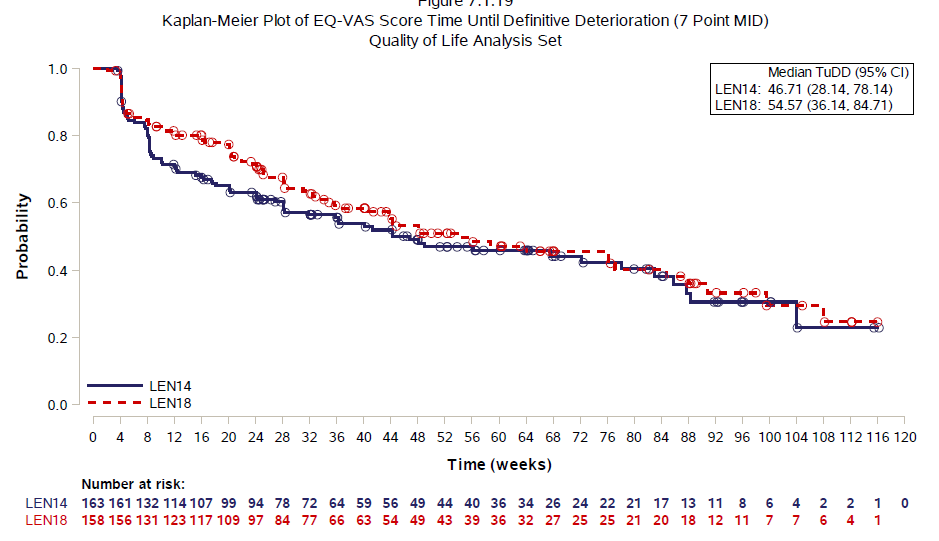

Supplement: oyac142_suppl_Supplementary_Material [file oyac142_suppl_supplementary_material.docx]
